# Peripheral shift in the viable chondrocyte population of the medial femoral condyle after anterior cruciate ligament injury in the porcine knee

**DOI:** 10.1371/journal.pone.0256765

**Published:** 2021-08-26

**Authors:** Meggin Q. Costa, Martha M. Murray, Jakob T. Sieker, Naga Padmini Karamchedu, Benedikt L. Proffen, Braden C. Fleming

**Affiliations:** 1 Department of Orthopaedics, Warren Alpert Medical School of Brown University/Rhode Island Hospital, Providence, RI, United States of America; 2 Department of Orthopaedic Surgery, Boston Children’s Hospital, Harvard Medical School, Boston, MA, United States of America; University of Rochester, UNITED STATES

## Abstract

Anterior cruciate ligament injuries result in posttraumatic osteoarthritis in the medial compartment of the knee, even after surgical treatment. How the chondrocyte distribution within the articular cartilage changes early in this process is currently unknown. The study objective was to investigate the chondrocyte distribution within the medial femoral condyle after an anterior cruciate ligament transection in a preclinical model. Forty-two adolescent Yucatan minipigs were allocated to receive unilateral anterior cruciate ligament surgery (n = 36) or no surgery (n = 6). Central coronal sections of the medial femoral condyle were obtained at 1- and 4 weeks after surgery, and the chondrocyte distribution was measured via whole slide imaging and a cell counting batch processing tool utilized in ImageJ. Ki-67 immunohistochemistry was performed to identify proliferating cells. Empty lacunae, karyolysis, karyorrhexis, and pyknosis were used to identify areas of irreversible cell injury. The mean area of irreversible cell injury was 0% in the intact controls, 13.4% (95% confidence interval: 6.4, 20.3) at 1-week post-injury and 19.3% (9.7, 28.9) at 4 weeks post-injury (p < .015). These areas occurred closest to the femoral intra-articular notch. The remaining areas containing viable chondrocytes had Ki-67-positive cells (p < .02) and increased cell density in the middle (p < .03) and deep zones (p = .001). For the entire section, the total chondrocyte number did not change significantly post-operatively; however, the density of cells in the peripheral regions of the medial femoral condyle increased significantly at 1- and 4 weeks post-injury relative to the intact control groups (p = .032 and .004, respectively). These data demonstrate a peripheral shift in the viable chondrocyte population of the medial femoral condyle after anterior cruciate ligament injury and further suggest that chondrocytes with the capacity to proliferate are not confined to one particular cartilage layer.

## Introduction

Healthy cartilage is noted to have relatively few chondrocytes embedded in a dense extracellular matrix [1]. Chondrocyte proliferation and death are relatively infrequent in normal cartilage [2]. In contrast, osteoarthritis (OA) is marked by both chondrocyte loss [3,4] and chondrocyte proliferation, largely noted as “cloning” on histologic sections of the diseased cartilage and thought to occur in the superficial zones of the cartilage during the early stages of OA [5]. The progression from a healthy, relatively stable cellular tissue to the arthritic condition is not well understood. This knowledge gap is due in part to the challenges associated with obtaining samples for study from patients in the early stages of the disease.

Large animal models have proven useful for studying the progression of posttraumatic OA (PTOA), particularly after an anterior cruciate ligament (ACL) injury [6–8]. Small animal models have shown that chondrocyte loss occurs in the early stages of PTOA [9–11], and that a subset of chondrocytes maintains the ability to proliferate at these early, post-injury time points [12,13]. However, the size of the joint and articular cartilage thickness of small animal models are much less than those of humans [14]. Recently, a study conducted in the adolescent Yucatan minipig model reported that chondrocytes in the medial femoral condyle exhibit a gene-expression signature consistent with chondrocyte proliferation at 1- and 4 weeks after ACL injury [8]. A systematic evaluation of early changes in chondrocyte density and proliferation within the medial femoral condyle at the early time points following an ACL injury in a large animal model would provide insight into the early cartilage changes associated with PTOA initiation.

To measure these potential effects, we developed a new histomorphometric approach to count all cells within a complete coronal section of the cartilage of the medial femoral condyle instead of focusing on a specific region of interest as has been done previously [15]. The reason the medial femoral condyle was selected is that previous studies have shown that the medial compartment is most affected in both humans [16–18] and pigs [6] following ACL injury. By combining whole slide imaging with a cell counting batch processing tool utilizing ImageJ, we were able to quantify the cell count and cell density within each whole section to measure the cell distribution patterns throughout the width and depth of the cartilage.

The objective of this investigation was to determine the changes in viable cell density and distribution throughout the medial femoral condyle at early time points after ACL injury. We utilized quantitative histopathological techniques on medial femoral condyle articular cartilage tissues obtained at 1- and 4 weeks following an ACL injury and surgery in adolescent Yucatan minipigs [7,8]. These time points, joint region, and preclinical model were selected for this investigation, as a microscopic PTOA stage has been previously shown to occur in the medial femoral condyle within 4 weeks of an ACL transection in the Yucatan minipig model [6]. Furthermore, the Yucatan minipig is an established model of PTOA that has been shown to produce both macroscopic cartilage damage and non-cartilage OA features within one year [6,19] that are consistent in geographic extent and severity with the human condition at 10–20 years after ACL injury [20]. Justifications for the use of this model are further expounded upon in a supplement (S1 Methods). We hypothesized that areas with irreversible chondrocyte injury, as well as chondrocyte proliferation, would occur following a surgically induced destabilizing knee injury, and that the chondrocyte count and distribution at 1- and 4 weeks post-injury would differ from an intact control group. Given that different surgical interventions were used, the potential surgical treatment effects (ACL transection, ACL reconstruction, vs. bio-enhanced ACL repair) on these parameters were also evaluated and reported as a supplement (S1 Table).

## Methods

### Study design

Articular cartilage tissues were acquired from a previously published large animal experiment with cross-sectional assessments at two post-surgery time points [7,8]. Institutional Animal Care and Use Committee approval (Brown University IACUC# 16080000221) was obtained prior to initiating this study, and the study was performed under the ARRIVE guidelines [21]. Forty-two adolescent (ages 13–18 months) Yucatan minipigs (Sinclair BioResources, Columbia, MO) were allocated to receive unilateral anterior cruciate ligament (ACL) transection surgery (n = 36) or no surgery (Intact, n = 6). Of those animals receiving ACL transection surgery, outcome assessments were performed at 1-week (1w, n = 18) or 4 weeks (4w, n = 18) after surgery. Within each time point, 6 animals were allocated to no further treatment, 6 to immediate ACL reconstruction surgery and 6 to immediate bio-enhanced ACL repair surgery. This repair procedure uses a bioactive scaffold to enhance ACL healing, as previously described [6]. Both the ACL reconstruction and the bio-enhanced repair procedures were included in this investigation to allow outcome comparisons between these two groups, as previous studies in the Yucatan minipig model have shown that the degree of macroscopic cartilage damage is greater in the ACL reconstruction group relative to the bio-enhanced ACL repair group [6,22]. A computer-based random permutation stratified for sex determined each animal’s group allocation and side of surgery with an equal number of males and females in each group. A detailed description of the surgical procedures have been previously published [8]. Briefly, a medial arthrotomy and fat pad resection were performed to expose the ACL while the animal was under anesthesia [6,8]. The ACL was then transected between the proximal and middle thirds of the ligament, and a clinical exam was performed to verify complete ACL transection [6,8]. In the animals assigned to receive ACL reconstruction surgery, a fresh-frozen bone-patellar tendon-bone allograft, which was harvested from an age-, weight-, and sex-matched donor, was implanted as previously described [6,8]. In animals assigned to bio-enhanced ACL repair surgery, an extracellular matrix scaffold in combination with autologous blood was implanted as previously described [6,8]. The sample size (n = 42) was a sample of convenience as the study was powered to address hypotheses related to protein expression levels in cartilage and synovium [7,8]. A summary of the procedures and detailed information regarding animal husbandry and pain management are available in the supplement (S1 Methods).

Following euthanasia, a coronal plane osteochondral slab of 5 mm thickness was obtained immediately anterior to the center of the condyles, defined as the middle of the anterior-posterior dimension of the medial condyles. Histologic sections from these slabs were used to assess the articular cartilage using 7 measures of cell loss, proliferation, count and distribution (area of irreversible cell injury; Ki-67 expression; cell density (3 zones); total cell count; and mean median peripheral cell position).

### Sample processing

Samples were fixed in 10% neutral buffered formalin for 48 hours, followed by decalcification in 10% Formic acid (EMD Millipore, Darmstadt, Germany)/5% Formalin solution (AcrosOrganics, Belgium) for 10 days at room temperature on a shaker table as previously described [8]. The solution was replaced every 48 hours. Samples were dehydrated in 70%, 95% and 100% Ethanol, 1:1 Ethanol/Xylene and 100% Xylene at room temperature for 24 hours each, immersed in paraffin at 60˚C for 48 hours and then embedded. Per specimen, at least two 6 μm sections were mounted on silanized microscope slides (Superfrost Plus, Thermo Scientific, Waltham,MA); one of which was stained with nuclear fast red staining for subsequent quantitative cell counting and distribution analyses, and the other was stained to evaluate the immunolocalization of Ki-67, a well-established cell proliferation marker used in previous OA studies [13,23–26].

The nuclear fast red staining solution was prepared using distilled water, 5% of Aluminum sulfate and 0.1% of nuclear fast red. Deparaffinized cartilage sections were stained for 10 minutes, washed, dehydrated and cover-slipped with organic mounting medium.

**Fig 1:**
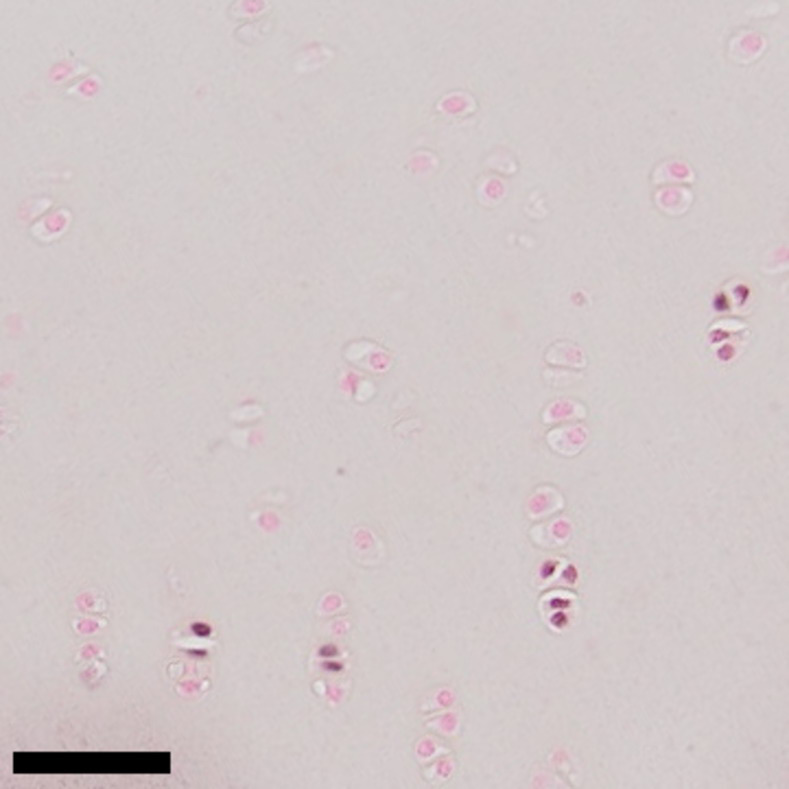
Immunohistochemical staining of chondrocytes with nuclear fast red counterstain for detection of Ki-67 expression. Chondrocytes with darker nuclei are positive for Ki-67 expression. Bar indicates 50 micrometers.

For the immunolocalization of the cell proliferation marker Ki-67, deparaffinized cartilage sections were heated to 60˚C in Antigen Unmasking Solution (H-3301, Vector Laboratories, Burlingame, CA) for 14 hours. Samples were washed in running distilled water for 5 minutes before and after antigen retrieval. After washing in phosphate buffered saline (PBS) at pH 7.2 with Azide (MB-011, Rockland, Limerick, PA) for 5 minutes, unspecific binding sites were blocked using 2.5% horse serum (ImmPRESS™ AP Anti-Rabbit IgG Polymer Detection Kit, MP-5401, Vector Laboratories) at room temperature for 20 minutes. The primary antibody, recombinant rabbit monoclonal Anti-Ki67 IgG antibody (SP6, ab16667, Abcam, Cambridge, MA), was diluted in buffer with 2.5% normal horse serum. Specimens were incubated with Anti-Ki67 at room temperature for 12 hours, followed by three 5-minute washes in Wash-Buffer (PBS pH 7.0 with Azide [MB-011, Rockland] and 0.1% Tween 20 [Vector Laboratories]). For detection, the specimens were incubated with an anti-rabbit IgG-alkaline phosphatase conjugate (MP-5401, Vector Laboratories) for 30 minutes at room temperature, followed by three 5-minute washes in Wash-Buffer and incubation in substrate solution (Vector® Black Substrate Kit, Alkaline Phosphatase, SK-5200, Vector Laboratories) for 30 minutes. Specimens were counterstained with nuclear fast red and a cover slip was applied (Fig 1).

Whole slide images were obtained using an Olympus VS120 Virtual Microscopy System with a 20x objective. Staining and whole slide imaging were performed in one batch and with identical microscope settings. The stored images from the scope were converted to “tif” format and downscaled by a factor of 2 using FIJI for further analysis [27].

### Outcome measures

Cell loss was assessed using the area of irreversible cell injury (%), quantified as the area of articular cartilage with signs of irreversible cell injury as a percentage of the total articular cartilage area. Karyolysis, pyknosis, karyorrhexis and empty lacunae were considered features of irreversible cell injury [28,29].

**Fig 2:**
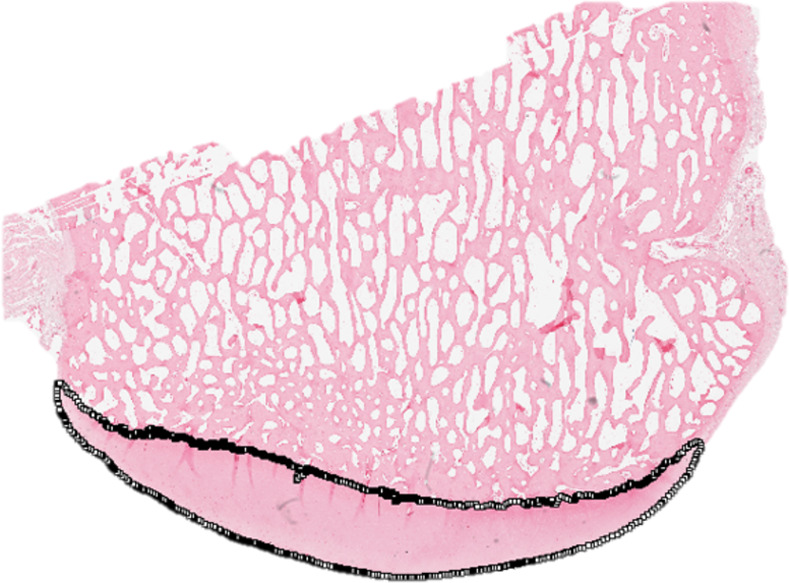
Oriented histologic section from 5 mm thick osteochondral slab and selected region of interest for viable chondrocyte quantification. The region of interest was manually selected in FIJI using the segmented line tool, as demonstrated in this figure.

**Fig 3:**
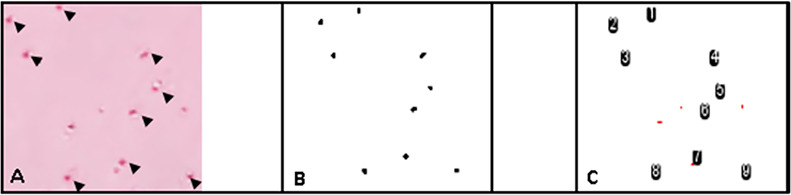
Viable chondrocyte selection and quantification in FIJI using the Threshold Colour plugin. A) Subset of nuclear fast red stained chondrocytes. Black arrows indicate viable chondrocytes. B) Thresholded, viable chondrocytes. Note the elimination of non-viable chondrocytes and extracellular matrix. C) Count of thresholded, viable chondrocytes.

Cell proliferation was assessed indirectly in two ways: the expression of the proliferation marker protein Ki-67 (encoded by *MKI67*) in full-thickness articular cartilage samples [30], and by testing for differences in cell density between the two time points. The superficial zone, middle zone and deep zone cell density values were assessed and compared separately. For each zone, the density was reported as cells/mm^2^. Ki-67 expression was quantified based on the *MKI67* mRNA using RNA-seq in combination with site matched articular cartilage samples [8]. The cell density measurements were performed in areas with preserved zonality and without irreversible cell injury. The superficial zone was defined as the area from the articular surface to a depth of 170 μm, while the deep zone was defined as the area extending 170 μm up from the subchondral bone plate. The middle zone was defined as the remaining cartilage area between the superficial and deep zones [31]. For each zone, the number of nuclei was measured using FIJI and the Threshold Colour plugin (Threshold Colour v1.12a G. Landini 27/Sep/2010). Articular cartilage matrix and background were eliminated using a saturation threshold (45–255 pass). Debris and artifacts were eliminated by filtering for particle size (10-1400px pass). Chondrocytes in clusters were separated using the watershed function. A second filter to eliminate debris and artifacts was applied based on size and circularity (10-400px pass, circularity of 0.4–1.0 pass). Collectively, these filters excluded cells with characteristics of karyolysis, pyknosis, karyorrhexis and empty lacunae; the remaining particles were considered viable nuclei and counted (Figs 2 and 3). The zone-specific cell count was then divided by the area of the respective zone to yield the superficial zone cell density, middle zone cell density and deep zone cell density.

The net effects of cell loss and proliferation on the cell count and cell distribution were assessed using the total cell count and the mean central-peripheral cell position, respectively. The total cell count was measured as described above using the entire cartilage area as the region of interest (including areas with irreversible cell injury). To determine the mean central-peripheral cell position, all images were rotated and mirrored (if applicable) to align the aspect of the cartilage surfaces adjacent to the intercondylar notch on the left margin of the image (corresponding to relative x-position of 0) and the peripheral aspect of the cartilage surfaces on the right margin of the image (corresponding to relative x-position of 100) (Fig 2). Nuclei were detected as described above and x-axis positions were recorded and used to determine the mean cell position (ranging from 0 to 100, with 0 corresponding to the medial and lateral aspects of the articular cartilage, respectively).

### Statistical analysis

To determine if significant differences in areas of irreversible cell injury, chondrocyte counts and distributions exist between the intact control group and the groups at 1- and 4 weeks post-surgery, the Kruskal-Wallis test was used, followed by Dunn’s post-hoc test with Holm’s p-value adjustment to correct for comparisons of multiple groups. Holm’s adjusted p-values < .05 were considered statistically significant. The identical approach was used to test for differences in chondrocyte density by cartilage zone (i.e., superficial, middle, and deep) between the intact control and the post-injury groups, as well as for differences in chondrocyte proliferation (i.e., Ki-67 expression) in the full-thickness cartilage samples. In addition, the above methods were used to test for differences between surgical treatment groups at each time point for all outcome measures. All statistical analyses were performed using R version 3.2.3 (The R Foundation for Statistical Computing).

## Results

At both post-surgery time points, there were no significant differences in any of the assessed cartilage outcomes between groups with ACL reconstruction, bio-enhanced ACL repair or untreated ACL transection surgery (S1 Table). The surgical group results were thus pooled for each time point and are presented below.

**Fig 4:**
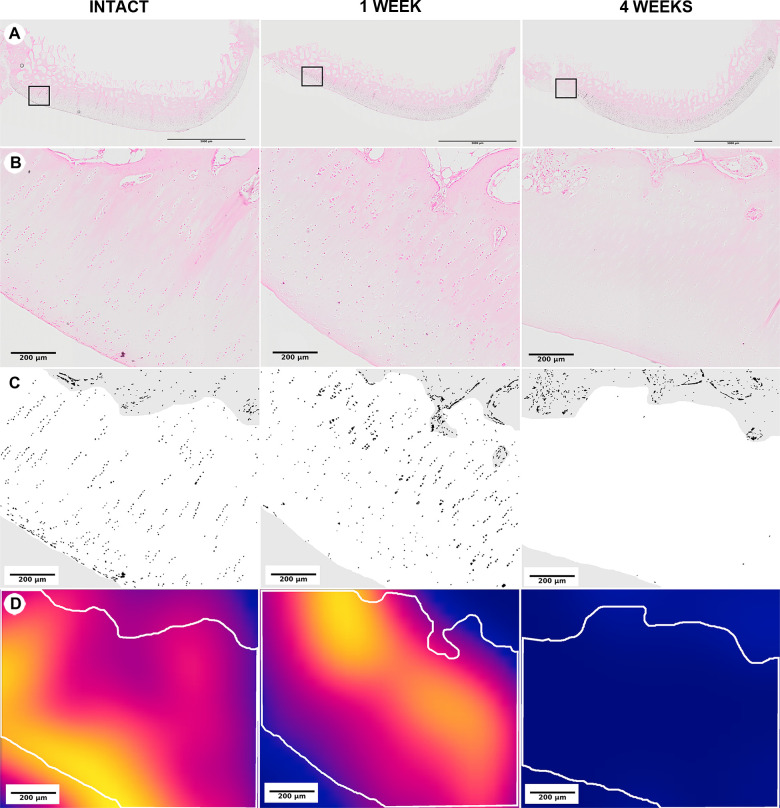
Medial femoral condyle articular cartilage irreversible cell injury and loss at 1- and 4 weeks following surgical induction of PTOA. The magnifications shown in this figure were selected for illustrative purposes; all analyses were performed using a 20x objective. All sections are in the frontal plane and the area adjacent to the intercondylar notch is to the left of each figure. (Row A) Low magnification, H&E. Boxes indicate regions shown at higher magnification in Row B. (Row B) Higher magnification, H&E. Note the changes in chondrocyte distribution in areas articulating with the tibial spine at 1- and 4 weeks post-surgery. (Row C) Pseudocolored images of the lateral half of the areas in Row B highlighting the nuclei detected through image processing in black. (Row D) Heatmaps of chondrocyte cell density for the areas in Row C ranging from yellow–highest cell density, to red–intermediate cell density, to dark blue–no cell density. Note the loss of viable chondrocytes in this region for the 4-week specimens.

The area of irreversible cell injury values (%), the area of articular cartilage with signs of irreversible cell injury as a percentage of the total articular cartilage area, were significantly greater at 1- and 4 weeks post-injury than in intact controls (p = .013 and .004, respectively) (Fig 4). The mean area of irreversible cell injury was 13.4% (95% confidence interval: 6.4, 20.3) at 1-week post-injury and was greatest, 19.3% (9.7, 28.9), at 4 weeks post injury. No microscopic damage was identified in intact controls, i.e., 0% (0.0, 0.0).

**Fig 5:**
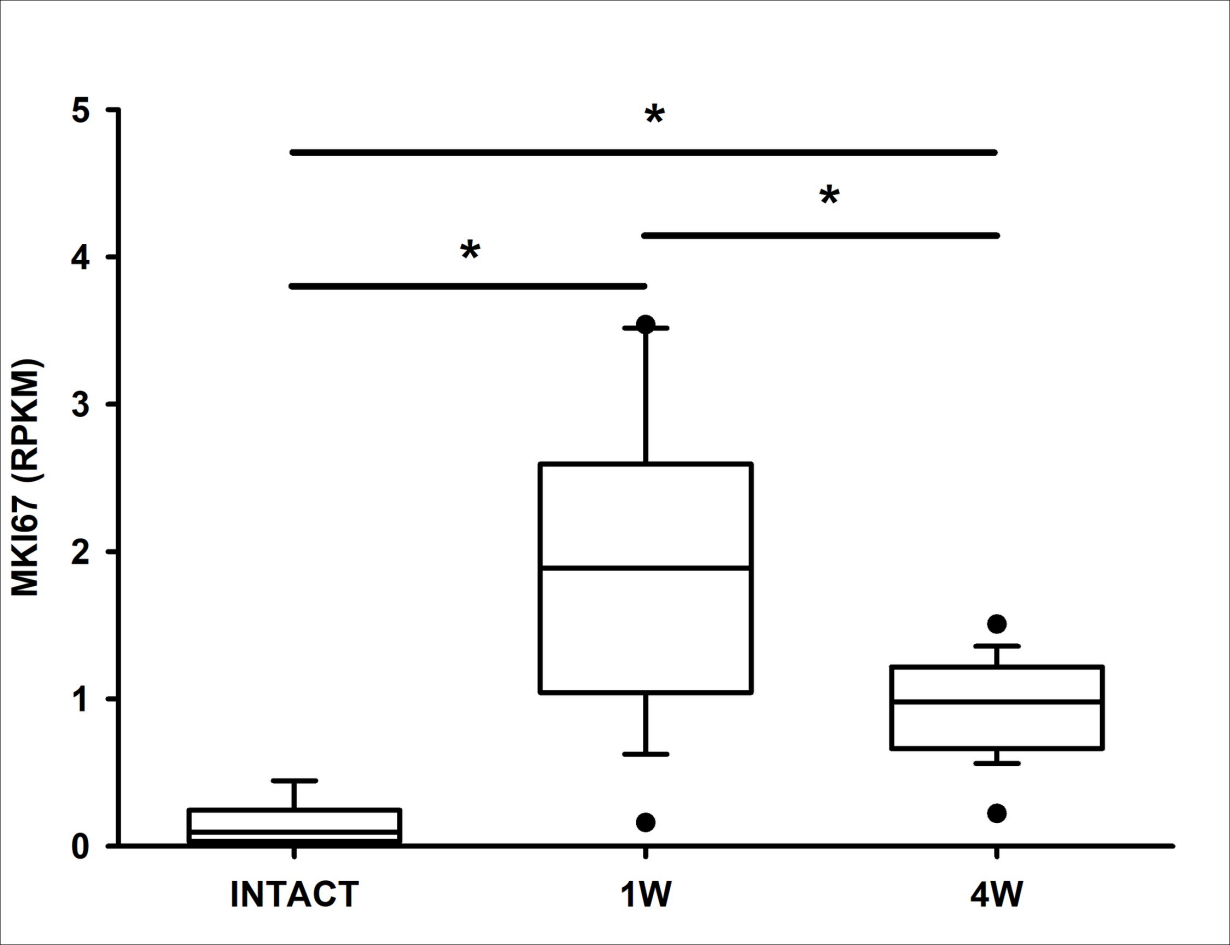
*MKI67* expression in reads per kilobase of transcript per million mapped reads across the intact and 1- and 4-week post-injury groups. Black bars and asterisks highlight group comparisons with statistically significant differences.

For the remaining cartilage with viable cells, the median Ki-67 expression levels (i.e., chondrocyte proliferation) were significantly greater at 1- and 4 weeks post-injury compared to intact controls (p < .001 and .017, respectively) (Fig 5). Ki-67 expression peaked 1-week post-injury and then dropped but remained elevated at 4 weeks. Ki-67 expressing cells were found in the superficial, middle and deep zones.

**Fig 6:**
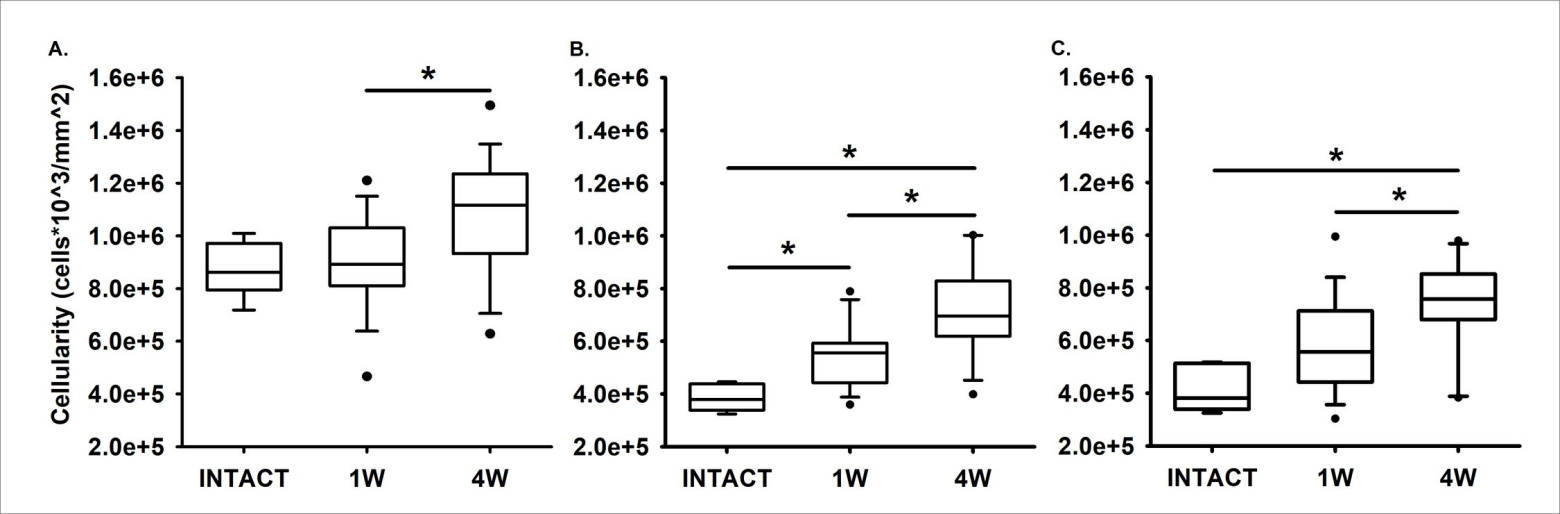
Changes in viable chondrocyte cellular density over time in the superficial (A.), middle (B.), and deep zones (C.) of articular cartilage. Black bars and asterisks highlight group comparisons with statistically significant differences.

The superficial zone cell density values were not significantly different at 1- and 4 weeks post-injury compared to the intact controls (p = .637 and = .057) (Fig 6A). However, the middle zone cell density values were significantly greater at 1- and 4 weeks post-injury than in intact controls (p = .029 and < .001, respectively). The middle zone cell density was greatest at 4 weeks post-injury (Fig 6B). The deep zone cell density was significantly greater at 4 weeks post-injury relative to intact controls (p = .001), but not at 1-week post-injury (p = .051) (Fig 6C).

**Fig 7:**
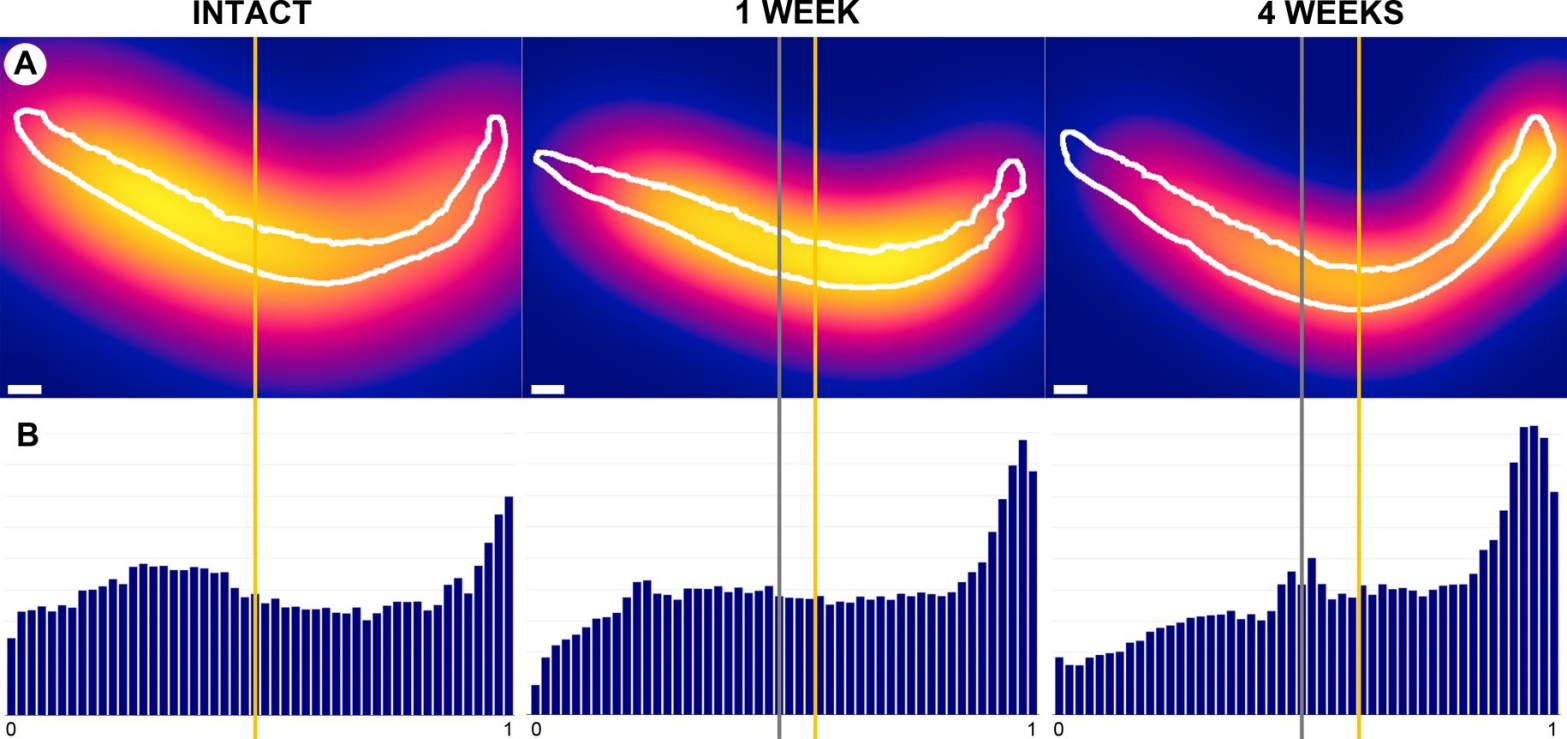
Medial femoral condyle articular cartilage demonstrates a shift in cell distribution away from areas articulating with the tibial spine at 1- and 4 weeks following surgical induction of posttraumatic OA. The tibial spine is to the left for each image. (Row A) Heatmaps of chondrocyte cell density, ranging from yellow–highest cell density, to red–intermediate cell density, to dark blue–zero cell density. White bars indicate 1mm. (Row B) Histograms showing the relative frequency of nuclei by relative peripheral position from 0-notch to 1-peripheral. The gray lines indicate the center, and the yellow lines indicate the mean median peripheral chondrocyte position for each specimen. Note the lateral shift of the median cell position over time.

The total cell count values were not significantly different between post-injury and intact control groups. The mean (95% confidence intervals) total cell count at 4 weeks post-injury was 9,198 cells (7674, 10721) as compared to 7,926 cells (7250, 8602) in intact controls. Concomitantly, the mean median peripheral cell position values (%) were significantly higher at 1- and 4 weeks post-injury than in intact control groups (p = 0.032 and p = .004, respectively). The mean peripheral cell position peaked 4 weeks post-injury at 61.3% (57.1, 65.5), corresponding to an increase in cell density in the peripheral regions of the medial femoral condyle (i.e., away from the intra-articular notch), when compared to intact values of 50.0% (48.1, 52.0) (Fig 7).

## Discussion

The results of this study demonstrate that chondrocyte loss and proliferation have a significant net effect on the cell distribution within the articular cartilage of the medial femoral condyle following ACL surgery, while not significantly changing the cell number in the whole cartilage section. Specifically, an increase in cell density in the peripheral region of the medial femoral condyle was observed, consistent with predominant cell loss in the cartilage areas nearest the tibial spine. Cell proliferation was increased in the more peripheral cartilage. Furthermore, based on the molecular and morphological markers, cell proliferation was also present in the middle and deep zones of the articular cartilage, suggesting that chondrocytes with the capacity to proliferate were not confined to one particular cartilage zone.

The area of irreversible cell injury, as indicated by karyolysis, pyknosis, karyorrhexis and empty lacunae, increased from 13% of the cartilage section width at one week to almost 20% of the cartilage section width at four weeks post-injury, supporting our initial hypothesis. This finding suggests that chondrocyte death may be dependent on time from injury, although it remains unclear whether early chondrocyte death is a cause or product of OA pathogenesis. The present study, however, suggests that it may be neither, as the three experimental groups did not produce significantly different chondrocyte outcomes despite one of the groups (bio-enhanced ACL repair) going on to have significantly less macroscopic OA at the one year time point [6,32]. This finding may also be partly because the differences within 4 weeks may be too subtle to detect. Future work is needed to resolve this question.

The area of irreversible cell loss was found primarily near the intra-articular notch of the medial femoral condyle. Other studies of OA patterns after ACL transection have also reported cell loss in the medial femoral condyle [33,34], consistent with our findings. In the current study, the observed distribution of irreversible cell areas throughout the cartilage thickness (Fig 4) suggests that chondrocytes undergoing cell death were present in all three cartilage zones, also consistent with prior reports [2,35]. These findings suggest that chondrocyte death may be a driving event in OA pathogenesis, which is largely consistent with prior studies of cell death in OA [28]. Importantly, the rate of cell death observed in the present study approximates the rates of chondrocyte apoptosis previously observed in human OA, which range from <1% to over 40% [36–39]. However, the predominant form of cell death involved in OA remains unclear. Future investigations are therefore needed to determine the relative contributions of distinct modes of cell death at different OA stages and in different regions of the knee joint.

The finding that proliferating chondrocytes were present in the middle and deep zones as early as one week after the induction of OA was unexpected. Prior studies in normal and later stage osteoarthritic samples have suggested that chondrocyte proliferation during OA development is initially limited to the superficial and upper-middle cartilage zones and then progresses to the middle and deep zones only in late or end stage OA cartilage [5,39]. The current study involved cartilage in an injured joint that had not yet gone on to moderate OA, so was neither normal nor in an advanced disease state. This very early disease state is difficult to sample in clinical trials, which may explain why this finding has not been noted before. The presence of the proliferating cells in the deeper cartilage zones may be attributable to a local, less differentiated progenitor cell population [40] or due to the migration of a superficial cell population [41,42]. Further studies are necessary to determine the source of these proliferating cells.

There are several study limitations to consider. It is unclear how this study in a large animal quadruped model will translate to human disease. Another limitation is the use of late adolescent animals, which might increase the probability of detecting proliferating cells compared to adults [43]. However, ACL injuries are most prevalent in the adolescent population [44], and place these patients at greater risk for posttraumatic OA [45]. Therefore, this is an important age group to study. An additional limitation is the small group sample size, which may have reduced the power of the study for detecting differences between the treatment groups (S1 Table). Nonetheless, significant differences were found relative to the control group. In addition, it is unclear whether the irreversible cell injury observed in this study was due to OA development induced via ACL transection or if this finding was the result of unintended surgical effects, including exposure to atmospheric conditions or the effect of an arthrotomy on the synovial tissue response. However, the ACL transection method in this model has been shown to reliably produce macroscopic cartilage damage and non-cartilage features that are consistent with the human OA condition after ACL injury [6,20,22], thus supporting the use of this model to study PTOA. It is also important to note that the cell counting batch processing tool used in this study is novel and has not yet been applied in other models. However, the ability of this technique to assess changes in cell count over time supports its use in the porcine model. Finally, the cell densities and distributions were only assessed in the cartilage of the medial femoral condyle, which may limit the generalizability to the whole joint; however, the medial femoral condyle has been shown to present the greatest macroscopic damage in the ACL injured and reconstructed knee both in humans [16–18] and in pigs [6,17]. Therefore, this location was selected for study. As only one 5mm thick medial femoral condylar cartilage section was assessed per knee, future studies may need to examine multiple planes to better understand the behavior of the entire medial femoral condyle chondrocyte population at early time points post-injury.

In conclusion, the area of irreversible cell injury following the surgical induction of early posttraumatic OA in the late adolescent porcine knee increased by almost 20% in the medial femoral condyle within 4 weeks of surgery and was found in the more central aspect of the knee. Cellular proliferation was found peripherally and in all zones of the cartilage. While the overall number of cells in the cartilage section did not change over time, these data demonstrate an increase in the viable and proliferating cell population in the peripheral region of the medial femoral condyle after ACL injury and suggest that chondrocytes with the capacity to proliferate are not confined to one particular cartilage layer.

## Supporting information

S1 Checklist The ARRIVE guidelines checklist. (PDF)

S1 Table Outcomes by treatment group. Cell density measurements exclude areas of irreversible cell injury; One 4-week sample was too disrupted to define the superficial, mid, and deep zones and was excluded from the analyses of these three measures.
(PDF)

S1 Methods A summary of the surgical procedures and detailed information regarding animal husbandry and pain management. (PDF)
