# Supplementary material for: Peripheral shift in the viable chondrocyte population of the medial femoral condyle after anterior cruciate ligament injury in the porcine knee
Source: PLoS One. 2021 Aug 26;16(8):e0256765. doi: 10.1371/journal.pone.0256765 (PMC8389427; doi:10.1371/journal.pone.0256765)
Supplement: S1 Table — Cell density measurements exclude areas of irreversible cell injury; One 4-week sample was too disrupted to define the superficial, mid, and deep zones and was excluded from the analyses of these three measures. (PDF) [file pone.0256765.s002.pdf]

Table S1. Outcomes by treatment group

	Intact	1 week			4 weeks			1 week			4 weeks		
	**Intact** (n=6) *Mean (95% CI)*	**Transection** (n=6) *Mean (95% CI)*	**Reconstruction** (n=6) *Mean (95% CI)*	**Repair** (n=6) *Mean (95% CI)*	**Transection** (n=6) *Mean (95% CI)*	**Reconstruction** (n=6) *Mean (95% CI)*	**Repair** (n=5-6) *Mean (95% CI)*	**Reconstruction vs. Transection** *P value*	**Repair vs. Transection** *P value*	**Repair vs. Reconstruction** *P value*	**Reconstruction vs. Transection** *P value*	**Repair vs. Transection** *P value*	**Repair vs. Reconstruction** *P value*
Area of irreversible cell injury (%)	0.0 (0.0, 0.0)	14.1 (-1.5, 29.8)	20.0 (7.2, 32.9)	5.9 (2.6, 9.3)	16.2 (2.7, 29.6)	24.6 (2.2, 46.9)	17.3 (2.1, 32.5)	1.000	1.000	1.000	1.000	1.000	1.000
Ki-67 expression (RPKM)	0.14 (0.02, 0.27)	1.45 (0.77, 2.13)	2.29 (1.29, 3.29)	1.87 (1.22, 2.52)	0.90 (0.57, 1.23)	0.94 (0.61, 1.28)	1.00 (0.81, 1.2)	1.000	1.000	1.000	1.000	1.000	1.000
Superficial zone cell density (cells/mm2)	872 (789, 955)	796 (594, 998)	932 (836, 1029)	965 (867, 1063)	1183 (1044, 1322)	998 (792, 1204)	1039 (848, 1231)	1.000	1.000	1.000	1.000	1.000	1.000
Mid zone cell density (cells/mm2)	385 (342, 427)	455 (417, 494)	661 (584, 739)	513 (440, 587)	704 (579, 828)	707 (570, 845)	736 (550, 922)	0.350	1.000	0.964	1.000	1.000	1.000
Deep zone cell density (cells/mm2)	410 (341, 480)	429 (375, 483)	730 (607, 854)	598 (456, 740)	759 (669, 849)	704 (553, 856)	752 (571, 933)	0.183	1.000	1.000	1.000	1.000	1.000
Total cell count	7926 (7250, 8602)	8369 (5509, 11229)	9385 (7742, 11028)	7991 (6512, 9469)	11327 (9687, 12967)	7741 (5896, 9585)	8525 (5042, 12008)	1.000	1.000	1.000	0.367	1.000	1.000
Mean mediolateral cell position (%)	50 (48.1, 52)	58.7 (50.2, 67.2)	57.3 (51.8, 62.8)	54.9 (52.5, 57.3)	63.5 (57.7, 69.4)	60.4 (53.6, 67.1)	60.1 (50.5, 69.6)	1.000	1.000	1.000	1.000	1.000	1.000

Cell density measurements exclude areas of irreversible cell injury; One 4-week sample was too disrupted to define the superficial, mid, and deep zones and was excluded from the analyses of these three measures.
