# Supplementary material for: Peripheral shift in the viable chondrocyte population of the medial femoral condyle after anterior cruciate ligament injury in the porcine knee
Source: PLoS One. 2021 Aug 26;16(8):e0256765. doi: 10.1371/journal.pone.0256765 (PMC8389427; doi:10.1371/journal.pone.0256765)
Supplement: S1 Methods — (PDF) [file pone.0256765.s003.pdf]

SUPPLEMENTAL METHODS

Animal model

The adolescent Yucatan minipig model was selected for this study as it exhibits various features of human knee joints.[1] The Yucatan minipig has been shown to develop macroscopic cartilage lesions consistent with post-traumatic osteoarthritis (OA) within one year following ACL transection.[2] The cartilage damage typically develops on the medial femoral condyle with more pronounced damage at areas adjacent to the tibial spine,[2] consistent with the damage observed in human patients following ACL reconstruction surgery.[3] Furthermore, the Yucatan model has also been shown to develop other non-cartilaginous features of post-traumatic OA, such as an early synovitis along with accompanying changes in protein markers of extracellular matrix breakdown.[4] The genetic[5] and pharmacokinetic[6] similarities between the porcine model and humans further support the use of the Yucatan minipig ACL transection model to study post-traumatic OA. The sample size (n=42) was a sample of convenience as the study was powered to address hypotheses related to protein expression levels in cartilage and synovium.[7,8]

Housing and husbandry

Following delivery to the animal care facility, all animals underwent a minimum of a 7-day quarantine and stabilization period. The pigs were housed in single cages (a minimum of 22.5 ft^2) with wood chips over the concrete floor. All pigs were housed in pens that were adjacent to pens housing other pigs. They were allowed to ambulate at all times. Animals were fed at several scheduled times per day. However, food was withheld a minimum of 12 hours before surgery and before euthanasia. No animals were excluded from the study and no modifications to the approved protocol were necessary over the course of the study.

Anesthesia

Anesthesia was induced using Telazol (4 mg/Kg) and Xylazine (2 mg/kg) followed by Propofol (3-7 mg/kg) and then maintained with Isoflurane (1-3 MAC) following intubation. Eyes were protected using an eye lubricant. Both limbs were shaved and scrubbed with Chlorhexidine and 70% alcohol until visibly clean, followed by a ten-minute evaporation period. Hoofs were covered with unsterile gloves. Animals were then transferred into the adjacent operating room, placed supine on a heating mat, and secured on the operation table. Animal health and anesthesia depth were maintained by monitoring respiratory rate, oxygen saturation, electrocardiogram, blood pressure, and body temperature. The surgical limb and ipsilateral lower body were then scrubbed three times using Betadine. Hoofs were covered with a sterile glove and secured using a sterile elastic wrap. One layer of sterile towels was placed around the surgical area, followed by a layer of sterile drapes, leaving only the surgical limb exposed during the procedure.

Prior to euthanasia, anesthesia was induced and maintained similar to that used for the surgical procedures. Animals were euthanized during deep anesthesia using an intravenous injection of a solution containing pentobarbital sodium and phenytoin sodium (Beuthanasia-D, 0.1ml/kg). Death was confirmed by the absence of blood pressure and heart sounds prior to obtaining the tissue samples.

Analgesia and Peri-operative care

Drug	Dose		Route	Frequency of application (times/day)	Duration (days)
	mg/kg	ml			
Buprenorphine	0.01		Intramuscular	Once, pre-op	1
Fentanyl Patch	2ug/kg/hr		Transdermal	Once, pre-op	3
Ceftiofur	5		Intramuscular	Once, pre-op	1
0.5% Bupivicaine + 2% Lidocaine		1.0	Subcutaneous around wound	Once, post-op	1
Ondansetron	4		Intramuscular or Intravenous	Once, post-op	1
Tylenol elixir	10-15		Orally	Every 6 hours	As needed

REFERENCES

1. Proffen BL, McElfresh M, Fleming BC, et al. 2012. A comparative anatomical study of the human knee and six animal species. The Knee 19:469-476.

2. Murray MM, Fleming BC. 2013. Use of a bioactive scaffold to stimulate anterior cruciate ligament healing also minimizes posttraumatic osteoarthritis after surgery. Am J Sports Med 41:1762-1770.

3. Okafor EC, Utturkar GM, Widmyer MR, et al. 2014. The effects of femoral graft placement on cartilage thickness after anterior cruciate ligament reconstruction. J Biomech 47:96-101.

4. Sieker JT, Ayturk UM, Proffen BL, et al. 2016. Immediate administration of intraarticular triamcinolone acetonide after joint injury modulates molecular outcomes associated with early synovitis. Arthritis Rheumatol 68:1637-1647.

5. Wernersson R, Schierup MH, Jorgensen FG, et al. 2005. Pigs in sequence space: a 0.66X coverage pig genome survey based on shotgun sequencing. BMC Genomics 6:70.

6. Swindle MM, Makin A, Herron AJ, et al. 2012. Swine as models in biomedical research and toxicology testing. Vet Pathol 49:344-356.

7. Sieker JT, Proffen BL, Waller KA, et al. 2018. Transcriptional profiling of synovium in a porcine model of early post-traumatic osteoarthritis. J Orthop Res 36:2128-2139.

8. Sieker JT, Proffen BL, Waller KA, et al. 2018. Transcriptional profiling of articular cartilage in a porcine model of early post-traumatic osteoarthritis. J Orthop Res 36:318-329.
